# Solar Radiation and Vitamin D: Mitigating Environmental Factors in Autoimmune Disease

**DOI:** 10.1155/2012/619381

**Published:** 2012-01-11

**Authors:** Gerry K. Schwalfenberg

**Affiliations:** Department of Family Medicine, University of Alberta, Suite No. 301, 9509-156 Street, Edmonton, AB, Canada T6G 2M7

## Abstract

This paper looks at the environmental role of vitamin D and solar radiation as risk reduction factors in autoimmune disease. Five diseases are considered: multiple sclerosis, type 1 diabetes, rheumatoid arthritis, autoimmune disease of the thyroid, and inflammatory bowel disease. Clinical relevant studies and factors that may indicate evidence that autoimmune disease is a vitamin D-sensitive disease are presented. Studies that have resulted in prevention or amelioration of some autoimmune disease are discussed. An example of the utility of supplementing vitamin D in an unusual autoimmune disease, idiopathic thrombocytic purpura, is presented.

## 1. Introduction

After cardiovascular disease and cancer, autoimmune diseases, taken as a group, are the third leading cause of morbidity and mortality in the industrialized world [1]. There are more than eighty defined autoimmune diseases [2] known in humans. A multifactorial interaction between genetic predisposition, immunologic, hormonal and environmental stimuli contributes to the development of autoimmune disease [3]. Agents that may trigger autoimmune disease include infections, vaccine immunogens, adjuvants used to increase immune response, smoking and stress, and so forth as outlined in the literature [4]. (See Table 1). The prevalence of some autoimmune diseases may be as high as 5% in the general population [5]. Little is known about mitigating factors until recently. Evidence that autoimmune disease may be a vitamin D-sensitive disease comes from many studies. Solar radiation (UVR) and vitamin D have been shown to inhibit the induction of a number of autoimmune diseases in animal models [6–8]. (See Table 2). Autoimmune disease should vary by season, temperature, level of ultraviolet irradiance, latitude, race or skin color, BMI, physical activity, and vitamin D supplementation, if it is a vitamin D-sensitive process. 

**Figure 1:**
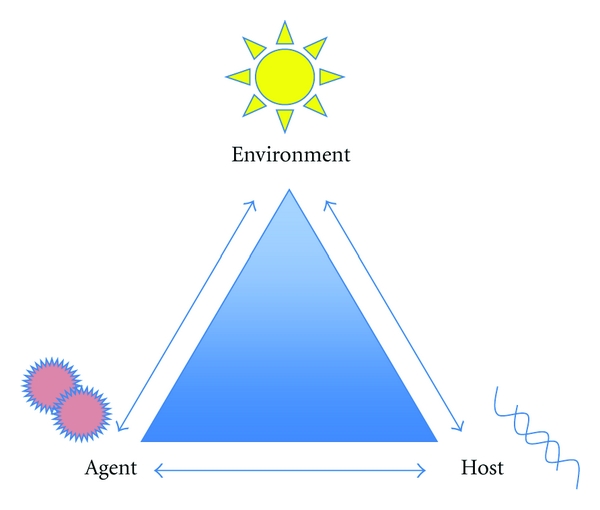
Autoimmune disease causation triangle. Adapted from [9]. Used with permission.

This paper will discuss the interaction between the host, the agent, and the environment as depicted in Figure 1. The environmental factor being considered is UVB radiation induced vitamin D or supplemental vitamin D. Five autoimmune diseases will be discussed. Multiple sclerosis, type 1 diabetes, rheumatoid arthritis, autoimmune disease of the thyroid, and inflammatory bowel disease.

At the end of this paper, an example of the utility of vitamin D in idiopathic thrombocytic purpura, another autoimmune disease, is discussed.

## 2. A Brief Overview of Vitamin D and Its Potential Role in Autoimmune Disease

Vitamin D deficiency is common in latitudes far from the equator [10, 11], and solar abstinence has been in vogue for the past few decades for fear of inducing skin cancer. Sun exposure (a minimal erythemal dose with full body exposure) can rapidly produce 10,000 or more units of vitamin D [12], and toxicity has not been ascribed to this method of achieving normal vitamin D status. Vitamin D used orally at 10,000 IU a day for several months does not cause toxicity [13]. Vitamin D is a secosteroid hormone available in some foods and supplements or produced in the skin from 7-dehydrocholesterol after exposure to ultraviolet B light. The resulting previtamin D is then hydroxylated in the liver to hydroxyvitamin D (25(OH)D) and further hydroxylated in the kidney to 1,25-dihydroxyvitamin D (1,25(OH)_2_D) which is the active hormone involved in calcium absorption in the gut. Circulating 25(OH)D (which is considered the measure of vitamin D adequacy) may also be used as substrate in many cells to locally produce (1,25(OH)_2_D), the active hormone, via the CYP27B1 (1*α*-hydroxylase) enzyme and is inactivated by the CYP24A (24-hydroxylase) enzyme [14]. The classical role of vitamin D is to regulate calcium homeostasis [15]. Short latency diseases, such as rickets and osteomalacia, can be cured with 25(OH)D levels > 25 nmol/L. In a long-latency disease, such as osteoporosis, levels of 25(OH)D > 50 nml/L have been shown to reduce fractures.

In the last twenty years, the importance of vitamin D in the role of a hormone has been shown to influence numerous other diseases including cancer by increasing apoptosis in cancer cells and protecting DNA in normal cells [16]. Its effect on the immune system and infections is only beginning to be understood, and much higher doses of vitamin D may be needed to be effective in combating viruses, bacteria, and fungi [17]. Vitamin D is now recognized to be crucial in beta-defensin production in Crohn's disease [18].

The role in regulating the immune system in regards to self-tolerance and autoimmunity begins with an understanding of the impact of vitamin D on our genes. Research is showing that there are 2776 “binding sites” on the human genome to which vitamin D attaches, with at least 229 genes associated with Crohn's disease and type 1 diabetes [19]. Many of the sites are concentrated around genes linked to autoimmune conditions as described in this paper. Beyond this, vitamin D suppresses autoimmune disease pathology by regulating differentiation and activity of CD4+ T cells resulting in a more balanced T1/T2 response favoring less development of self-reactive T cells and autoimmunity [2].

## 3. Multiple Sclerosis

In regards to the host, it is known that only 30% of monozygotic twin pairs eventually get multiple sclerosis (MS) leading us to believe that exposure to one or more environmental risk factors is necessary for the development of MS [20]. Recently, two large genomic studies have confirmed the unambiguous associations with the DRB1 and DQB alleles of the human leukocyte antigen class II region and susceptibility to MS [21].

Multiple environmental factors may play a role in multiple sclerosis. An example is the Epstein-Barr virus infections [22]. The risk of developing multiple sclerosis following infectious mono is increased significantly for more than 30 years following infection [23]. Even after 10 years following infection, the risk has been defined as at least four-fold [24]. Sunlight and vitamin D may be protective, and MS demonstrates vitamin D sensitivity [25].

Evidence shows that MS correlates positively with higher latitude, with latitudes > 37.5 degrees from the equator having significantly higher rates of MS [26–29]. The time of the first exacerbation after disease onset shows seasonal variation with 76% of exacerbations occurring in winter [30]. However, in another study, the likelihood and intensity of MS disease activity correlated positively with spring and summer (March to August) along with increased temperature and UVR [31]. Relapses of MS have a biphasic pattern with peaks in early spring when vitamin D levels are low and late fall when levels are declining [32]. MS also correlates positively with season of birth, with a significantly higher incidence of MS in those born in May, corresponding to low vitamin D levels in the winter months prior to giving birth [33]. As well, MS correlates inversely with altitude, with higher elevation (>2000 Meters) receiving more intense solar radiation having lower rates of MS [34]. Adiposity has been associated with lower vitamin D levels [35, 36], and a higher body mass index (BMI) has been associated with higher incidence of MS in adolescent women but not in adult women [37]. Multiple sclerosis correlates positively with skin color where sun avoidance is more likely (fair skin phenotype) [38]. In Norway, the intake of vitamin D in costal communities is estimated to be three times the average intake of those living inland and this is inversely associated with MS [39].

There are only a limited number of trials currently available in humans. An American study of more than 187,000 women followed for 10–20 years showed promising results with females taking at least 400 IU of supplemental vitamin D daily. The risk of developing MS was decreased by 40% [40]. Vitamin D_3_ has been used safely in MS patients at high doses from 28 to 280, 000 IU per week. Mean levels of 25(OH)D rose to a mean of 385 nmol/L without causing hypercalcemia after being given the highest dose. Disease progression and activity were not affected in this study, but the number of gadolinium-enhancing lesions per patient assessed by nuclear magnetic brain scan was significantly reduced [41]. The highest dose in this study was only used for the last 6 weeks of the study, and longer-term use at this dose may risk significant toxicity in some patients. A trial using high-dose vitamin D_2_ to achieve 25(OH)D levels of 130–195 nmol/L did not reduce MRI lesions in relapsing remitting multiple sclerosis [42]. In a recently reported trial using escalating doses up to 40,000 IU of vitamin D_3_ for 28 weeks followed by 10,000 IU daily for 12 weeks, there were no significant adverse events and there appeared to be significantly less progression of disability in the treatment group [43].

## 4. Type 1 Diabetes

As with multiple sclerosis only about 34% of identical twins will develop type 1 diabetes [44]. Thus, exposure to other environmental stimuli must play an important role.

In the past rubella infection has been implicated as an inducer of type I diabetes. Several studies now indicate infection with enteroviruses seem to be linked to the induction of islet cell destruction and development of autoantibodies [45, 46]. Stress in the mother during pregnancy has been associated with elevated islet autoantibodies in cord blood [47].

The following is a summary of the evidence that type 1 diabetes is a vitamin D-sensitive disease. Type I diabetes varies by latitude with higher incidence in latitudes further from the equator (both north or south of the equator) except where local weather may influence this [48]. A yearly cyclical pattern (consistent over 20 years) of type 1 diabetes in Newfoundland reveals a peak incidence in winter [49]. Greater exposure to erythemal UVB radiation is negatively correlated with the incidence of type 1 diabetes [50]. The birth months of March to June correlate with an increased incidence of diabetes in Britain with prenatal exposure to low vitamin D levels during the winter months [51]. The average yearly temperature also correlates inversely with the incidence of type 1 diabetes [52]. Higher BMI as well as lower plasma levels of 25(OH)D correlate directly with development of type 1 diabetes in young adults [53, 54]. Maternal vitamin D levels have been shown to correlate inversely to the presence of islet autoantibodies in the offspring [55]. Supplementation of vitamin D in early childhood appears to reduce the chances of developing type 1 diabetes [56].

There is a large body of evidence showing that lack of vitamin D early in life is linked to the development of type 1 diabetes. Vitamin D used at a dose of 2000 IU in infants has been shown to reduced the subsequent development of type 1 diabetes over the next thirty years by 78 percent [57]. The use of cod liver oil in pregnancy and during the first year of life has been shown to reduce the risk of childhood onset type 1 diabetes. However, it was not possible to determine if vitamin D, omega-3 fatty acids, or the combination contributed to this result [58, 59]. The use of 400 IU of vitamin D has not been shown to reduce diabetes incidence [60]. In Finland the use of 2000 IU of vitamin D in children was recommended from 1964 to 1975 when it was lowered to 1000 IU of vitamin D supplementation with a small increase in type 1 diabetes incidence following this recommendation. A further reduction to the use of 400 IU of vitamin D in 1992 correlates with a significant rise in the incidence of type 1 diabetes since then [61]. A meta-analysis and systematic review on the use of vitamin D supplementation in early childhood in type 1 diabetes showed that the risk of developing type 1 diabetes was significantly reduced in infants with supplementation [56]. The pooled odds ratio was 0.71 (a CI of 0.60 to 0.84), and there was evidence of a dose-response effect showing a lower risk of developing type 1 diabetes with the use of higher amounts of vitamin D. In regards to sun exposure, the incidence of type 1 diabetes approaches zero in areas with high levels of UVB irradiance [48].

## 5. Rheumatoid Arthritis (RA)

The maximum genetic contribution indicated by studies on monozygotic twins is about 15% when it comes to rheumatoid arthritis (RA) and systemic lupus erythematosus (SLE) [62]. The HLA-DRB1 gene represents the major determinant of genetic predisposition to RA [63].

The association of infections and RA is still being debated in the literature. There is some evidence that RA-like diseases may result from the immunologic interaction between the host and bacterial peptidoglycans. Several agents relative to RA have been entertained such as heat shock proteins, bacterial IgG FC-binding proteins and rheumatoid factors [64]. Retroviruses and enteropathogenic bacteria continue to be intensively discussed candidates [65]. IgM antibodies to parvovirus B19 indicating recent infection correlate with juvenile idiopathic arthritis [66]. Certainly smoking has been cited as being a major risk factor both in RA and SLE as well as a risk factor for RF-positive and anti-citrulline antibody titers [67]. The risk only diminishes slowly after several years of cessation of smoking.

Increasing latitude in the northern hemisphere has been shown to correlate with increased risk of RA providing some support for a beneficial role of UVR [68]. Patients with undifferentiated connective tissue diseases have a seasonal variance in 25(OH)D levels, being significantly lower than controls in corresponding seasons [69]. Seasonal variations showed lower disease activity with higher 25(OH)D levels [70]. It appears that rheumatoid arthritis does not have association with month of birth [71]. Rheumatoid arthritis activity is inversely related to 25(OH)D levels [72]. Rheumatoid arthritis severity is associated positively with BMI with a high BMI having a greater risk of low 25(OH)D levels [73]. The incidence of arthritis in children is highest in Caucasian versus East Indian, First Nations as well as African American [74, 75]. The Iowa Women's Health Study showed an inverse association of vitamin D intake and rheumatoid arthritis [76] There was a 34% reduction in the development of rheumatoid arthritis with greater vitamin D intake. Women using a multivitamin with 400 IU of vitamin D reduced their risk of developing RA by 40% [76]. An open-label study using a high-dose vitamin D3 analogue resulted in improvement of symptoms in RA in 89% of patients with 45% of patients entertaining a complete remission [77].

## 6. Autoimmune Disease of the Thyroid

Autoimmune disease of the thyroid (AITD) is very prevalent with 5% of the population being affected. This includes both Hashimoto's (HT) and Graves' disease (GD) [5]. Several susceptibility genes for AITD have been identified, and it has been estimated that up to 80% may be attributable to genes [78]. However, the concordance rates in monozygotic twins is about 30–60%, while dizygotic twins is only 3–9%, and up to 50% of siblings of patients with GD having thyroid autoantibodies [79]. Thus, environmental factors contribute significantly to the expression of the disease.

Environmental factors are numerous and include iodine excess and deficiency, selenium deficiency, oral contraceptive use, parity, low birth weight, seasonal variation, allergy, smoking, radiation, viral and bacterial infections, and so forth [78]. Overall, data suggests that cumulative cigarette consumption significantly increases the risk of developing AITD [80]. Hashimoto's thyroiditis is associated with vitamin D insufficiency with AITD patients having significantly lower levels than controls [81, 82]. In AITD 72% of patients had 25(OH)D levels less than 25 nmol/L compared to 30.6% in healthy individuals. In patients with Hashimoto's thyroiditis, 79% had levels less than 25 nmol/L compared to 52% in normal controls.

The lowest incidence of AIDT disease was found in July to October (when vitamin D levels are high), and the highest incidence was in January to March when vitamin D levels tend to be low in the northern hemisphere [83]. The incidence of diagnosis of thyrotoxicosis is highest in May just after the winter when vitamin D levels are beginning to rise; however, this may also be a reflection of feeling hot with increasing ambient temperatures [84]. There is no information on altitude or temperature. Vitamin D levels are lower in AITD patients than in normal controls and supplementation is recommended [82]. There are presently no studies using vitamin D as an intervention in AITD; however, supplementation has been suggested.

## 7. Inflammatory Bowel Disease

In regards to genetic predisposition, a national German study has shown that concordance rates in monozygotic twins are about 35% for Crohn's disease (CD) and 16% for ulcerative colitis (UC), showing that environmental factors play a significant role in inflammatory bowel disease (IBD) [85].

An environmental trigger shown to increase the risk of development of CD is smoking. Many other triggers have been proposed such as infectious gastroenteritis, oral contraceptives, invasive *E*. *coli*, and antibiotics [86]. Vitamin D insufficiency is associated with inflammatory bowel disease [87, 88]. In another study 25(OH)D levels were lower in those with severe disease activity and less sun exposure [89].

A study in France has shown that there is an increasing incidence of UC with northern latitude [90]. Smaller studies did not show any seasonal pattern for IBD, but a larger study showed an increase incidence in those born in the first half of the year [91] while others show an increased incidence of exacerbations in summer. There is no information on altitude or temperature. Obesity is more common in Crohn's disease at the time of diagnosis [92]. Vitamin D deficiency is very prevalent in IBD [93] and the effects of low-dose supplementation are poor [94]. A clinical trial using 1200 IU of vitamin D_3_ for 12 months resulted in a reduced risk of relapse from 29% to 13%, although this did not quite reach statistical significance [95]. Other trials are currently under way.

A striking example in which vitamin D made a significant change in morbidity in another autoimmune disease follows.

## 8. A Case History and Discussion of the Benefit of Vitamin D in Idiopathic Thrombocytopenic Purpura in an Adult

Idiopathic thrombocytopenic purpura (ITP) is an autoimmune disease in which most patients have antibodies to specific membrane glycoprotein's on platelets. The incidence in adults is about 33/1,000,000 [96]of which about 10/1,000,000 become refractory. Spontaneous remission is uncommon in adults. The 5-year mortality rate is significantly elevated in adults over the age of 60 (47.8%) versus those below 40 years of age (2.2%), respectively. The most serious complication is hemorrhage of which intracranial hemorrhage is the most significant.

Vitamin D has been shown to improve outcomes and prevent some autoimmune diseases if taken early in life as discussed previously in the paper.

Treatment of ITP usually includes medications such as corticosteroids, splenectomy danazol, and various immune suppressant therapies [97]. The use of vitamin D_3_ in ITP has not been described in the literature.

This example describes a patient who had refractory ITP who has been treated in the past with a splenectomy, danazol, and prednisone rescue during intercurrent illness. A review of the history of this case revealed a 48-year-old female who was found to have a very low platelet count in 1998, which remained persistent over time. After consultation with a hematologist, the diagnosis of ITP was made. Her platelets continued to drop so she had a splenectomy, which improved her platelet count, but it never achieved normal. Despite the use of danazol, her platelet count never normalized. She had frequent episodes of low platelets as low as 8 × 10^9^ with intercurrent illnesses such as colds or flus. In 2006 she was found to have an inadequate level of 25(OH)D of 65 nmol/L. Her platelet count at that time was 8 × 10^9^ after a viral illness. She was treated successfully with a tapering dose of Prednisone. She was started on vitamin D_3_ 2000 IU daily after this episode and during the next two years while she was on this dose she did not have any flus or colds and her platelet count never fell below 44 × 10^9^and was usually from 70 to 80 × 10^9^. This was quite out of the normal for her since she had at least one low episode a year. After being on this dose for two years, a neighbor in her building where she resided suggested that she was going to become toxic on this dose and she stopped her vitamin D. About three months later, she again had an upper respiratory infection (URI) and her platelets dropped only to 50 × 10^9^ and she was started on prednisone with recovery of her platelets to 140 × 10^9^. She was seen after this, and it was recommended that she resume her vitamin D_3_ at a higher dose of 4000 IU daily. She was feeling quite well, and her platelets remained above 70 and continued to rise so she discontinued her danazol. Again, she had no further episodes of flu or colds for the next two years and her platelets did not drop below 70. Her vitamin D level on this dose was 88 nmol/L after 4 months. She phoned one day that she was sick with a URI, and it was suggested that she take 10,000 IU of vitamin D for a 3 days and have her platelets checked. She had her platelets checked after being on this dose for two days, and they were in the normal range much higher than they had been for years. The platelet count was 248 × 10^9^. Her vitamin D level was 99 nmol/L at this time. She continues on the 4000 IU vitamin D, and she continues off the danazol and remains well. Her latest platelet count was 318 × 10^9^.

This case presents a significant response to vitamin D_3_ in ITP. As well, it demonstrates a recurrent failure with lack of vitamin D and restoration of a normal platelet count on a higher dosing of vitamin D_3_, which did not result in toxicity. The vitamin D_3_ rescue with 10,000 IU of vitamin D_3_ appeared to result in a similar response as that of prednisone used in past treatments over the years. ITP has been shown to have spontaneous remission in some people; however, it is uncommon in older patients. Is it possible that restoration of vitamin D levels results in some of these cases of spontaneous resolution? At this time this is not known. Restoring vitamin D to a level that is safe appears to be sensible supportive therapy.

Restoration of adequate platelets has never been demonstrated with repletion of inadequate vitamin D levels in the literature. Certainly in this example, vitamin D_3_ restoring reasonable platelet levels and reducing the number of infections is most fascinating. The reduction in platelet levels with removal of vitamin D_3_ with restoration of normal levels with an increased dose of vitamin D_3_, as well as rescue with higher levels of vitamin D_3_, is furthermore more intriguing. More studies would be warranted to demonstrate the benefit of adequate 25(OH)D levels in ITP.

## 9. Discussion and Conclusion

This paper outlines a number of autoimmune diseases that show vitamin D sensitivity as provided by supplementation, UVR, or other factors. These factors have been summarized in Table 3. At this time the research on the role of vitamin D in autoimmune disease is not conclusive, and much of the data comes from epidemiologic or case control studies. However, the evidence is increasingly pointing towards a significant role of vitamin D in reducing the incidence and burden of autoimmune diseases.

The link between various autoimmune diseases and vitamin D in various ecological, population, and case control studies is summarized in Table 4. Interventional studies when available have been cited throughout this paper. Although not all linkages are positive in each disease, the ones that have been studied the most, such as MS and type 1 diabetes, are quite striking. Studies done in type 1 diabetes were done in infants, and supplementation during the prenatal and first year of life may result in a significant reduction of morbidity later in life. Vitamin D may very well be a significant factor in preventing the loss of tolerance to self and resultant autoimmune disease.

Population studies have shown consistently in many “northern” countries (i.e., Canada) that the majority of the population is vitamin D deficient. Knowing that a large proportion of the population do not have adequate levels of vitamin D, it may be prudent to restore levels to >100 nmol/L for optimum function of the immune system. 2000 IU of vitamin D_3_ has been shown to improve vitamin D status and achieve these levels in about 80% of patients [98]. This may decrease autoimmune disease incidence. Increasing vitamin D intake would benefit the general population with better bone health and prevention of cancer and infectious diseases. An estimate in the savings in healthcare expenditures with restoration of adequate vitamin D is in the billions of dollars [99, 100] These estimates include only a few of the many autoimmune diseases that vitamin D may mitigate. Further studies are warranted in addressing autoimmune disease and vitamin D. These studies should use vitamin D_3_ since, as in the above outlined studies, vitamin D_2_ was not effective and the latest evidence suggests that only vitamin D_3_, not vitamin D_2_, improves mortality [101].

## Figures and Tables

**Table 1:** Agents that trigger autoimmune disease.

Infections	Epstein-Barr's virus, cytomegalovirus, parvovirus, enteropathogenic bacteria
Vaccine immunogens	Multiple sclerosis, Guillain-Barre's syndrome, autism, rheumatoid arthritis, reactive arthritis, systemic lupus erythematosus, diabetes, vasculitis, dermatomyositis, polyarteritis nodosa
Adjuvants used to enhance immune response	Lupus erythematosus, brain directed autoantibodies, arthritis, nephritis
Birth control, pregnancy	Autoimmune thyroid disease
Smoking	Rheumatoid arthritis, systemic lupus erythematosus, multiple sclerosis, Graves' hyperthyroidism, Crohn's disease
Stress	Type 1 diabetes, Grave's disease

Adapted from [3, 4].

**Table 2:** Autoimmune diseases that are inhibited by 1,25(OH)2D in animal studies [6].

Autoimmune encephalomyelitis	
Collagen-induced arthritis	
Inflammatory bowel disease	
Type 1 diabetes	
Systemic erythematosus	
Thyroiditis	
Lyme arthritis	
Rheumatoid arthritis	
Multiple sclerosis	

**Table 3:** Autoimmunity and factors that relate to vitamin D-sensitive diseases.

Parameters relating to vitamin D	Multiple Sclerosis	Type 1 diabetes	Rheumatoid arthritis	Autoimmune disease of thyroid	Inflammatory bowel disease
Incidence seasonality	+	+	+	+	−
Seasonality of birth	+	+	−	+	+
Latitude	+	+	+	N/A	+
Altitude	+	N/A	N/A	N/A	N/A
Temperature	+	+	N/A	+	N/A
BMI	+	+	+	N/A	+
Race (skin tone)	+	+	−	N/A	N/A
UV radiance	+	+	+	+	+
Vitamin D intake	+	+	+	+	+

Evidence from studies listed in the paper for positive correlation of vitamin D-sensitive parameters in each disease. +: positive correlation, −: negative correlation, N/A: information lacking.

**Table 4:** Human studies in autoimmune disease.

Autoimmune disease	Study design (*N*)	Results
Multiple sclerosis	Prospective cohort studies NHS, NHS II supplementation of vitamin D (*N* = 187,365) [40]	40% reduction in developing MS with supplementation of 400 IU vitamin D
	Open label progressive supplementation of vitamin D (*N* = 12) [41]	The number of gadolinium-enhancing lesions was reduced
	Randomized control using 1000 IU versus 6000 IU daily of vitamin D_2_ for 6 months (*N* = 23) [42]	Vitamin D_2_ was not effective in reducing MRI lesions in RRMS
	Open-label randomized controlled trial (*N* = 49) [43]	8% in the treatment group had worsening disability versus 38% of patients in the control group
Diabetes	Birth cohort study (*N* = 12058) [57]	Use of 2000 IU had a reduced risk of developing diabetes by 78%
	Newly diagnosed diabetic children from 1980–2005 (*N* = 10737) [61]	Significant increase in incidence noted after reduction in vitamin D intake recommendation (decreased daily recommendation from 1000 IU to 400 IU)
	Meta-analysis of supplementation of vitamin D in infants [56]	29% reduction in risk of developing type 1 diabetes
Rheumatoid arthritis	Prospective cohort study dietary and supplement vitamin D intake (*N* = 29,368) [76]	34% reduction in developing RA in the supplement group > 400 IU vitamin D
	Open-label trial using high-dose oral alphacalcidiol therapy, (*N* = 19) [77]	Result in a positive effect on disease activity in 89% of patients
Autoimmune thyroid disease	None available to date	
Crohn's disease	Randomized double-blind placebo-controlled study (*N* = 94) [95]	1200 IU of vitamin D_3_ reduced the number of relapses in the treatment group by more than 50% during a 1 yr study
